# The Role of Acu-TENS in Hemodynamic Recovery after Open-Heart Surgery

**DOI:** 10.1093/ecam/neq015

**Published:** 2011-03-20

**Authors:** Maggie C. S. Ng, Alice Y. M. Jones, L. C. Cheng

**Affiliations:** ^1^Department of Physiotherapy, Grantham Hospital, China; ^2^Department of Rehabilitation Sciences, The Hong Kong Polytechnic University, China; ^3^Department of Cardiothoracic Surgery, Grantham Hospital, Hong Kong, China

## Abstract

Increased heart rate (HR) and reduced blood pressure (BP) are common consequences of cardiac surgery. This study investigated the effect of transcutaneous electrical nervous stimulation applied over acupuncture points (Acu-TENS) on HR, BP, rate pressure product (RPP) and nausea and vomiting score after open-heart surgery. After open heart surgery, 40 patients were randomly allocated to either an Acu-TENS group, which received a 40-min session of TENS applied bilaterally over the acupuncture point PC6 on postoperative days 1–5, or a Placebo-TENS group, which received identical electrode placement but with no electrical output from the TENS unit, despite an output indicator light appearing activated. HR, systolic and diastolic BPs (SBP and DBP) were recorded and RPP computed. Nausea and vomiting symptoms were quantified using a 4-point Likert scale before and after TENS intervention. Daily HR, BP and antiemetic administration data were recorded from a further 20 consecutive subjects who received no intervention and formed the Control group. A trend of decreasing HR and increasing BP in the Acu-TENS group was observed over the five postoperative days, with all variables returning to preoperative values by Day 4 (*P* > .2). In the Placebo-TENS and Control groups the HR remained higher (*P* < .0001), BP lower (*P* < .05) and RPP higher (*P* = .01) than respective preoperative values at Day 4. The dose of Maxolon required was lowest in the Acu-TENS group (*P* = .038). We concluded that Acu-TENS facilitated an earlier return to preoperative BP, HR and RPP values in patients after acute heart surgery.

An increase in heart rate (HR) is a normal sympathetic response to body trauma and a common consequence of major surgical procedures [1]. Any diminished cardiac contractility and/or perturbed peripheral vascular control may be reflected by hypotension in the postoperative period [2]. Impairment of baroreceptor reflex activity associated with vagal suppression [3] and derangement of autonomic regulation [4] are also phenomena commonly encountered in patients after cardiac surgery. A high resting HR is associated with increased myocardial work [5] that may be detrimental to patients with a marginal myocardial oxygen supply. HR regulation may be accomplished by pharmacological intervention but this may also be accompanied by unwanted cardiovascular and other body system effects [6].

Acupuncture is the placement of needles at specific points (acu-points) along body “meridians" (channels) through which internal energy (Qi) flows; acupuncture has been used in traditional Chinese medicine as a treatment to regulate HR [7]. Acupuncture proponents believe that stimulation of acu-points can restore the balance of Qi and facilitate recovery from bodily injury [8]. In western medicine, stimulation of certain acu-points is believed to be associated with stimulation of the autonomic nervous system and consequently affect HR [9, 10]. Effect of acupuncture on maintenance of BP in anesthetized and hemorrhagic dog models has also been reported [11, 12]. Acupuncture however is invasive and iatrogenic injuries have been reported [13, 14]. Transcutaneous electrical stimulation, on the other hand, is non-invasive and when applied over acu-points (Acu-TENS) has been shown to be associated with a faster return to resting HR and BP after exercise [15]. The effect of Acu-TENS on indices of post-surgical cardiac function has not been investigated.

Acupuncture is also believed to suppress the vomiting center in the mid-brain [16] and has been used as an alternative method of management for postoperative nausea and vomiting (PONV) [17, 18]. Exercise, being an important component of cardiac rehabilitation, is often prescribed with the aim of restoring cardiopulmonary fitness after cardiac surgery [19]. A high resting HR, low BP and symptoms of PONV are factors that limit the progression of a cardiac rehabilitation program. This study aimed to determine the effect of Acu-TENS on postoperative hemodynamic status and PONV symptoms after recent open-heart surgery.

## 1. Methods

**Figure 1:**
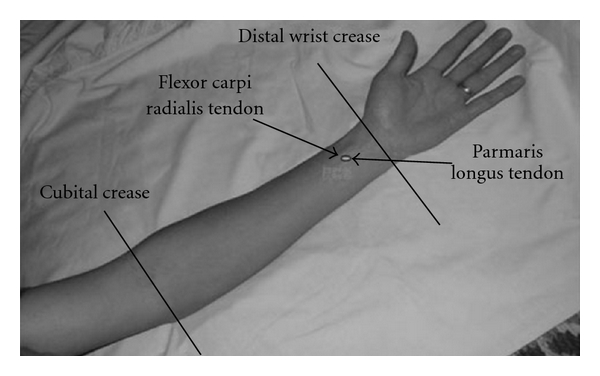
Location of the acupuncture point PC6.

Ethics approval was obtained from the Human Subjects Ethics Committees of the involved university and hospital. Written, informed consent was obtained from all patients prior to data collection. The study adopted a randomized, double-blind, placebo-controlled, clinical design. Randomization was achieved by a computer-generated sequencing protocol [20]. Subjects were randomly allocated to either the Acu-TENS or Placebo-TENS groups. Both groups received a 40-min intervention, at the same time on each postoperative day, 1–5. TENS electrodes were placed bilaterally on the acu-point PC6 (*Nei Guan*, at a point one-sixth of the distance between the wrist and the elbow, between the tendons of flexor carpi radialis and palmaris longus) (Figure 1). Patients in the Placebo-TENS group received no electrical output from the TENS unit despite the output indicator light appearing activated. Subjects in the Placebo-TENS group were told that the stimulation frequency was not perceivable by humans.

### 1.1. Participants

This study included patients who underwent elective open cardiac surgery, were successfully extubated on postoperative Day 1, transferred out of ICU on either postoperative Day 1 or Day 2, with stable BP, HR and normal ECG pattern, not requiring cardiac pacing, and who were able to follow verbal instructions. Patients not meeting the aforesaid criteria were excluded, together with those requiring medication outside routine dosage affecting HR, skin allergy to ultrasonic aqua gel and if vomiting prior to surgery. Patients with radial artery harvested for the coronary artery bypass graft were also excluded because the wound for radial artery harvest was adjacent to PC6.

### 1.2. Intervention Protocols

**Figure 2:**
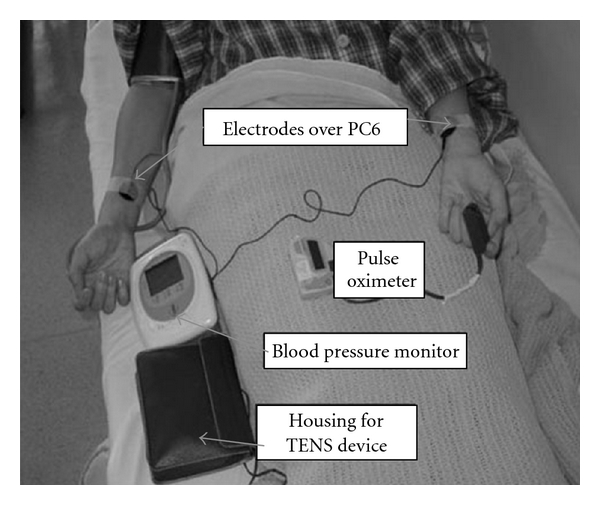
Electrode placements over PC6.

Patients were placed in a supine position and the skin area over the forearm was cleaned with an alcohol swab. The acu-point PC6 on each forearm was located and marked. Ultrasonic gel was applied to the electrode surface (1.5-cm diameter). The electrodes were then placed over the marked acu-points and secured by Micropore (3M) and attached to one of the two channels of a TENS machine (ITO-ES320 Japan) (Figure 2). The voltage output from the machine was validated and calibrated with an oscilloscope prior to data collection. Output to one of the channels, Channel 2, of the machine was disconnected. Subjects in the Acu-TENS group were attached to Channel 1 and received stimulation for 40 min at a frequency of 2 Hz, with a pulse duration of 0.2 ms. Patients were told that they would experience a tingling sensation from the stimulation and that they should endure the maximal intensity of the stimulation, without inducing pain. Patients in the Placebo-TENS group were attached to Channel 2 and were told that the frequency used for effective stimulation was outside the range of human sensory perception.

### 1.3. Outcome Measurements

Prior to treatment, all subjects were asked to relax in the supine position for 15 min. HR (MINOLTA PULSOX-3i Japan) and BP (OSIM iBPM os-5000 Japan) were recorded at 5-min intervals during the intervention and ECG changes were monitored continuously throughout the treatment. The subject's nausea and vomiting symptoms before and after TENS intervention were recorded using the 4-point Likert scale (0 = none, 1 = nausea but no vomiting, 2 = retching, 3 = vomiting) adopted by the local hospital for documentation of the patient's emetic status. HR and BP were also recorded before and after each intervention session. To control for circadian influences on HR and BP, these two variables were recorded at 07:00, 15:00 and 23:00 h on the day prior to operation, and on Days 1–5 postoperatively, for comparison analysis.

The treatment intervention and data recording were conducted separately by two investigators. The investigator responsible for TENS application attached electrodes to either Channel 1 or 2 according to computer-generated randomization. This investigator then placed the TENS unit in a black box that concealed the channel number from the other investigator responsible for data recording. The data- recording investigator and the patient were both blinded to the intervention grouping.

To evaluate the effect of Acu-TENS and placebo-TENS compared to the effect of no intervention at all, HR and BP data, and the daily dosage of metoclopramide (Maxolon) administered for anti-emetic management were extracted from the medical records of a further 20 consecutive patients as a comparator Control group.

### 1.4. Sample-Size Calculation

A previous crossover design study [15] determined that the mean time (standard deviation) required for post-exercise HR to return to pre-exercise levels after Acu-TENS, continuous Acu-TENS and Placebo-TENS was 5.47 (3.03), 4.84 (3.37) and 9.44 (3.68) s, respectively. For our study to achieve 80% power and assuming 5% type I error using one-way ANOVA, a sample size of 10 subjects per group is required. Allowing for the possible high exclusion rate in post-cardiac patients requiring intensive care, a conservative sample size of 20 subjects per group was considered appropriate.

### 1.5. Data Analysis

Correlation coefficient and regression analysis were used to evaluate the accuracy of the power and voltage output of the TENS unit and an intra-class coefficient (ICC) [1, 3] was used to assess the reliability of the BP monitor.

One-way ANOVA was used to compare age, body mass index (BMI), ejection fraction (EF) and duration of operation between the three subject groups. The Mann-Whitney *U*-test was used to compare the between-group duration of ICU stay. The chi-square analysis was used to compare sex, type of operation and use of medication, while a chi-square cross-tab test was used to analyze the between-group differences in nausea and vomiting scores. Two-way repeated measures ANOVA was used to analyze the pre- and post-differences in HR, SBP and RPP between groups across time, while one-way repeated measures ANOVA was used to assess within-group differences in HR, SBP and RPP (rate pressure product) across time for each group. *Post hoc* analysis was used to compare the within-group variable differences between pre-op values and those taken at Day 4 after operation. A *P*-value of .05 was taken as the level of significance for all tests. All analyses were performed using the Statistical Package for Social Sciences (SPSS) for windows (version 13.0).

## 2. Results

**Figure 3:**
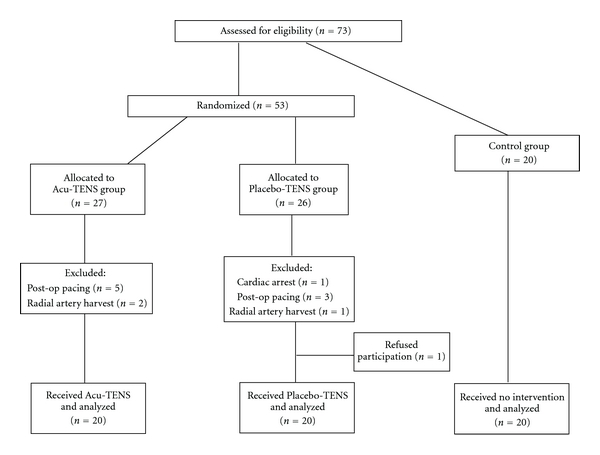
Consort diagram showing the flow of participants through the trial.

The output of the TENS machine (voltage, frequency and pulse width) was found to be stable and accurate. BP measurements were also found to be reliable (ICC = 0.99). A total of 73 subjects were recruited to the study. Thirteen subjects were excluded from the study (one had an intraoperative cardiac arrest, eight required postoperative pacing, three underwent radial artery harvesting and one refused to participate) (Figure 3). There were no differences in the demographic characteristics between subjects in the three groups (Table 1). 

### 2.1. Changes of HR, BP and RPP

**Figure 4:**
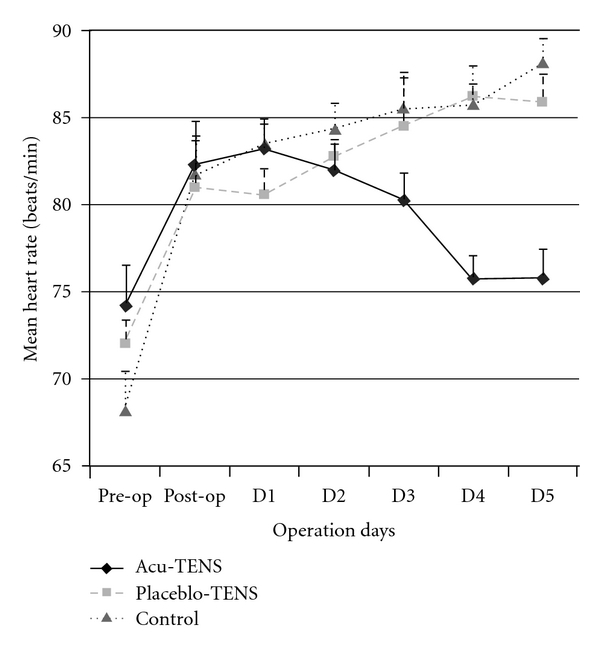
Mean resting heart rate at pre- and post-operation days.

Irrespective of the intervention group, all patients demonstrated an increase in resting HR on the post-operation day. For patients in the Placebo-TENS and Control groups, the resting HR, irrespective of the time of recording in the day, continued to increase through to post-op Day 4, and at Day 4, HR was still significantly higher than the pre-op values (*P* < .0001). Patients in the Acu-TENS group showed a decreasing resting HR trend (*P* = .007) that had returned to preoperative values by Day 4 (*P* = .053) (Table 2). The above pattern was consistent irrespective of the time of data collection (at 7:00, 15:00 and 23:00 h of the day).  Figure 4 illustrates the mean HR of the three groups postoperatively.

**Figure 5:**
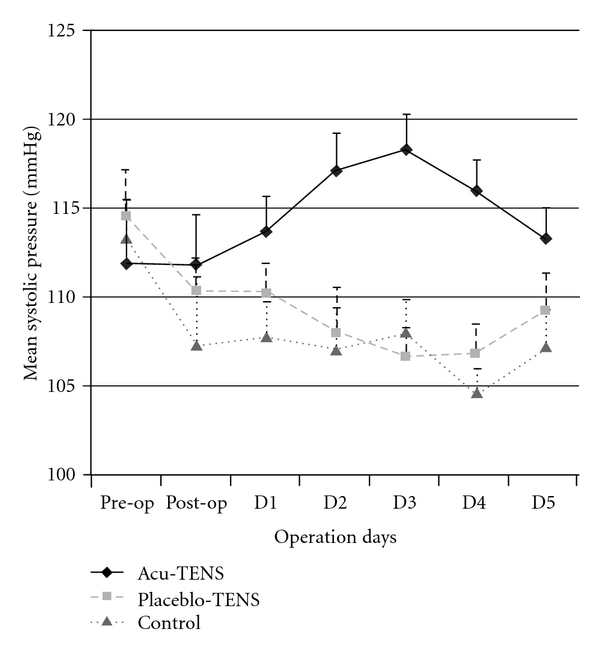
Mean SBP at pre- and post-operation days.

For patients in the Acu-TENS group, SBP increased over the first three postoperative days and returned to preoperative levels on Day 4 (*P* = .021), but for patients in the Placebo-TENS and Control groups, SBP decreased after operation and remained lower than preoperative values at Day 4 (*P* = .015 and .016, resp.). This pattern was consistent for the 7:00, 15:00 and 23:00 time points (Table 2, and Figure 5).

**Figure 6:**
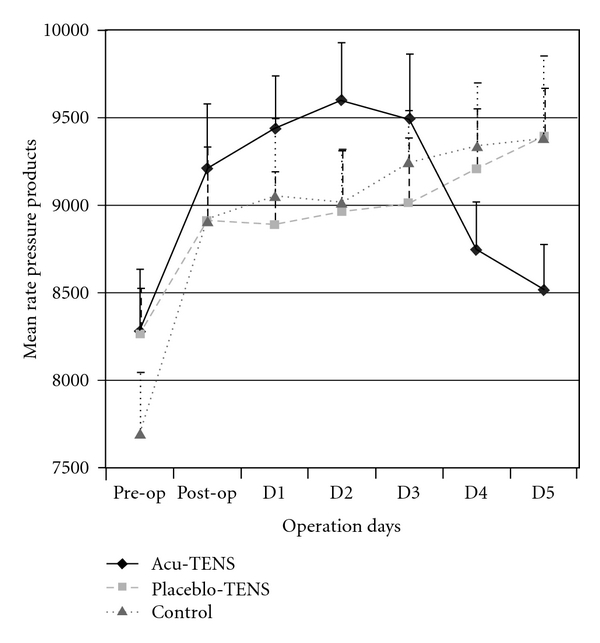
Mean rate pressure product at pre- and post-operation days.

The RPP in the Acu-TENS group followed a similar pattern of change as SBP, that is, increased during the acute postoperative period and returned to preoperative values at Day 4. The RPP of patients in the Placebo-TENS and Control groups increased after operation and remained significantly higher than the preoperative values at Day 4 (*P* = .008 and *P* < .001, resp.) (Table 2, and Figure 6). Again a similar data pattern was demonstrated for the three measurement time points.

### 2.2. Immediate Effect of the Intervention

This study showed a significant decrease in HR immediately after 40 min of Acu-TENS by 4.5 ± 0.49 bpm (*P* = .021), while the Placebo-TENS group demonstrated a mean change in HR of 0.79 ± 0.21 bpm (*P* = .0217). The mean between-group difference was −5.29 ± 0.37 (*P* = .000). A similar effect was demonstrated for RPP (*P* = .01). There was however no change in SBP immediately after Acu-TENS (*P* = .21) (Table 3). These data were not available for the Control group as they had no intervention. 

### 2.3. Changes in Nausea and Vomiting Symptoms

**Figure 7:**
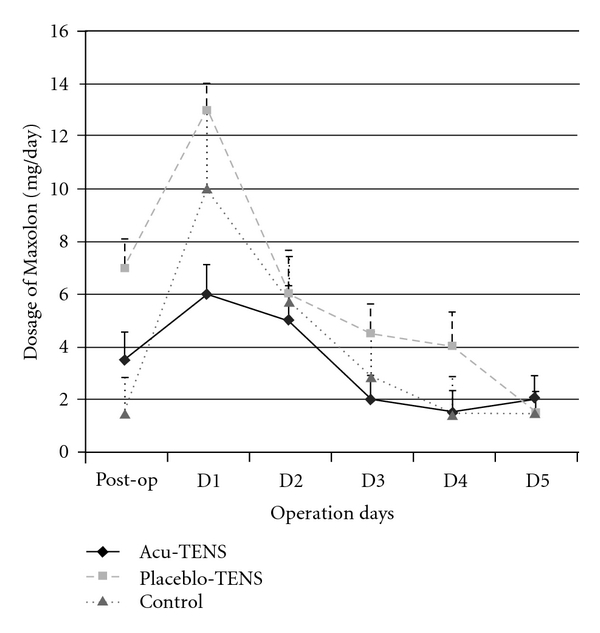
Daily postoperative antiemetic (Maxolon) administration.

The small number of patients in each group suffering nausea and vomiting limits the power of statistical analysis. The number of patients who scored 0 and 1 were therefore combined and labeled “mild" and those who scored 2 or 3 were combined to form the “severe" PONV group. The ratio of the number of patients scoring severe and mild PONV score was lower in the Acu-TENS group (1 : 19) compared to the Placebo-TENS group (4 : 16) (*P* < .001). The mean PONV scores before and after Acu-TENS and Placebo-TENS are displayed in Table 4. These data were not recorded in the Control group as there was no intervention. The dosage of Maxolon requested was lowest in the Acu-TENS group compared to the other two groups *P* = .038 (Figure 7). 

## 3. Discussion

In accord with previous reports, this study showed that resting HR increased after operation [3]. The present study demonstrated that application of Acu-TENS over acu-point PC6 for 40 min facilitated a faster return of resting HR to preoperative levels, maintained BP during the acute postoperative period and enhanced a quicker return of RPP, in patients after acute cardiac surgery. Furthermore, Acu-TENS was also associated with reduced symptoms of PONV with lower anti-emetic medication requirements.

Surgery causes an insult to the body, inducing responses such as pain, anxiety, pro-inflammatory reactions and increase in resting HR, all of which are modulated by the autonomic nervous system [1, 21]. Vagal suppression leading to increased HR has been described in patients after cardiac surgery [3] and depressed indices of autonomic function have been demonstrated in patients after coronary artery bypass graft [22]. HR can take up to 4 weeks to return to preoperative levels after coronary artery bypass grafting [4] and Soares et al. showed in their patient cohort that autonomic function reached lowest values 3–6 days after CABG and could take up to 60 days to return to pre-surgery values [22]. The rationale for deranged autonomic function seen after cardiac surgery and myocardial infarction has been extensively discussed [23–25]. Inhibition of the sympathetic tone in healthy volunteers has been shown to cause a reduction in amplitude and prolongation of latency of electrical responses, and slow HR [26]. Acupuncture stimulation of PC6 was associated with a reduction in resting HR and carbon dioxide production in healthy subjects [7, 18], allegedly due to autonomic nervous system modulation [27, 28]. Parasympathetic modulation of both HR and intestinal movement is largely mediated through the vagus nerve [18, 29]. TENS has been shown to inhibit the sympathetic skin response in healthy humans [30].

On the foregoing bases, we postulate that the faster return of the raised resting HR to preoperative levels in our Acu-TENS cohort was possibly due to induced vagal stimulation and/or inhibition of sympathetic neural activity through the acu-points PC6. The effect of electrical stimulation on hemodynamic responses has caused considerable debate in the literature [31]. Acupuncture is believed to have a “bi-directional" effect on BP. Stimulation of PC6 for 30 min has been shown to reduce both systolic and diastolic pressures (SBP and DBP) in patients with hypertension [32]; however, stimulation of PC6 for 1 h by electrical needle acupuncture was associated with a 40% increase in BP in anesthetized, thoracotomized dogs [11] and induced a 32% increase in end-systolic pressure in a hemorrhagic hypotensive dog model [12]. BP often falls in patients during the acute post-cardiac surgical period [2] and one aim of postoperative care in these patients is to maintain SBP between 110 and 140 mmHg [33]. This study demonstrated that patients in the Acu-TENS group maintained their SBP within this range during the acute postoperative period (Figure 5). The mean SBP of patients in the Placebo-TENS group however was below 110 mmHg during the same period. The higher RPP in the Acu-TENS group was likely to be the cause of the higher SBP during the first 3 days after operation; however, HR, RPP and BP all returned to preoperative levels by Day 4.

**Figure 8:**
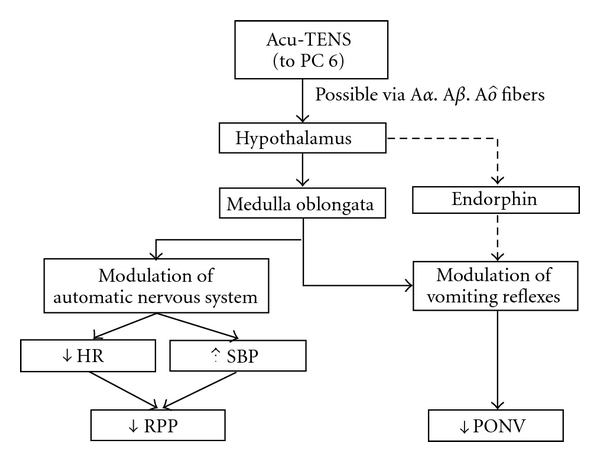
A proposed mechanism for Acu-TENS effect on hemodynamic parameters and postoperative nausea and vomiting score (HR = heart rate; SBP = systolic blood pressure; RPP = rate pressure product; PONV = postoperative nausea and vomiting score).

At the end of each Acu-TENS session, both HR and RPP decreased significantly. If Acu-TENS modulates the vagal nervous system, an “immediate" effect would be expected [18]. RPP reflects cardiac work and the immediate reduction in RPP suggests a possible myocardial protective effect through a decrease in myocardial contractility and lower myocardial oxygen demands. This is the first study to report the effect of acupuncture point stimulation on RPP. The early return of RPP to preoperative values suggests that Acu-TENS facilitates early recovery of hemodynamic parameters thereby conserving myocardial oxygen consumption in the early cardiac rehabilitation period.  Figure 8 illustrates a proposed mechanism for the hemodynamic effects of Acu-TENS. 

The effect of PC6 stimulation on the reduction in PONV and the reduced antiemetic requirements has been previously reported, but unlike this study, stimulation was via needle acupuncture [16, 18, 34, 35]. Proposed mechanisms of effect include endogenous (beta)-endorphin release in the cerebrospinal fluid, a change in serotonin transmission via activation of serotonergic and noradrenergic fibers [16] and vagal modulation that in turn affects intestinal responses [18]. Our study suggests that non-invasive TENS stimulation of PC6 was as effective as needle acupuncture in preventing PONV symptoms, possibly via the proposed pathways illustrated in Figure 8. In the literature, the stimulation of PC6 has been reported to be variously effective in suppressing nausea symptoms [36]. This may have much to do with the nature of the surgery in the different series [35]. In a study of 410 women after gynecological surgery, the incidence of PONV and the use of antiemetic medication were reduced from 46 to 33% after acupressure to bilateral PC6 [37]. Unilateral PC6 acu-point stimulation has been shown to reduce nausea, but not vomiting, after laparoscopic cholecystectomy [26] and electroconvulsive therapy [38].

### 3.1. Limitation of the Study

The relationship between Acu-TENS and vagal modulation could be further elucidated if HR variability was monitored during the application of Acu-TENS. Impedance cardiography and HR variability analysis were not available at the hospital during the time of data collection; however, this will be investigated in future studies.

## 4. Conclusion

This study showed that application of Acu-TENS to PC6 augmented BP and was associated with a faster return to preoperative resting HR and RPP in patients after recent open-heart surgery. Acu-TENS also reduced the postoperative symptoms of nausea and vomiting in this patient group. Acu-TENS is a non-invasive modality that does not require the presence of an acupuncturist and is easy to apply. This study suggests that Acu-TENS facilitates early recovery of hemodynamic variables and may reduce myocardial work, thereby providing a useful adjunctive therapy when optimizing postoperative cardiac rehabilitation.

## Figures and Tables

**Table 1:** Demographic data and baseline characteristics for the patient groups.

	Acu-TENS (*n* = 20)	Placebo-TENS (*n* = 20)	Control (*n* = 20)	*P*-value
Age	54.88 ± 3.90	53.05 ± 2.52	59.4 ± 2.28	.387
Body mass index	22.90 ± 0.75	22.14 ± 0.76	24.0 ± 0.92	.092
Ejection fraction	64.60 ± 1.52	60.40 ± 2.51	61.6 ± 1.65	.294
Duration of operation	4.05 ± 0.13	3.97 ± 0.15	4.22 ± 0.24	.945
Gender (M : F)	13 : 7	7 : 13	14 : 6	.500

Operation				
CABG : VR	9 : 11	5 : 15	12 : 8	.138
Metoprolol (mg day^−1^)	10.0 ± 2.81	5.00 ± 2.29	8.3 ± 2.63	.395
Amiodarone (mg day^−1^)	20 ± 13.75	20 ± 13.76	20 ± 13.72	.933
ACEi (mg day^−1^)	0	0.4 ± 0.28	0	.169
Digoxin (mg day^−1^)	37.5 ± 20.48	75 ± 26.28	25 ± 17.21	.244

Data are mean ± SE. M: male; F: female; CABG: coronary artery bypass graph; VR: valve replacement.

**Table 2:** Comparison of mean HR, SBP and RPP before and at postoperative Day 4.

	Acu-TENS group			Placebo-TENS group			Control group		
	Pre-op	Day 4	*P*-value	Pre-op	Day 4	*P*-value	Pre-op	Day 4	*P*-value
Heart rate (beat min^−1^)	74.2 (2.41)	75.8 (2.36)	.53	72.0 (1.43)	87.3 (1.82)	.000	63.4 (2.38)	83.03 (2.12)	.000
SBP (mmHg)	111.9 (3.64)	115.9 (2.94)	.21	114.6 (2.64)	106.9 (1.69)	.015	113.3 (3.95)	109.1 (1.93)	.016
Rate pressure product	8289.8 (358.9)	8740.9 (282.6)	.21	8261.6 (275.3)	9220.7 (352.0)	.008	7699.3 (353.5)	9344.5 (368.6)	.000

Data are mean ± (SE).

**Table 3:** Between group comparison of mean difference in HR, SBP and RPP before and after TENS intervention.

	Within-group difference		Between-group difference	Between-group *P*-value
	Acu-TENS	Placebo-TENS		
Heart rate (beat min^−1^)	−4.50 (0.49)	0.79 (0.21)	−5.29 (0.37)	.000
SBP (mmHg)	−4.95 (0.89)	0.35 (1.14)	−5.30 (0.85)	.206
Rate pressure product	−940.9 (123.9)	140.9 (123.6)	−1081.0 (115.2)	.010

Data are mean ± SE.

**Table 4:** Mean postoperative nausea and dyspnea score before and after Acu-TENS and placebo-TENS.

	Acu-TENS group	Placebo- TENS group	Between- group *P*-value
Pre-intervention score	0.23 ± 0.07	1 ± 0.19	.001
Post-intervention score	0.09 ± 0.05	0.85 ± 0.18	.000
Within group *P*-value	.034	.247	

Data are mean ± SE.
